# Association between suicide attempts and anemia in late-life depression inpatients

**DOI:** 10.1186/s12877-023-04649-9

**Published:** 2024-01-10

**Authors:** Jie Li, Lan Wang, Ziyi Wang, Fengxue Zhao, Yadi Sun, Ying Lu, Lei Yang

**Affiliations:** 1 The Second Affiliated Hospital, School of Nursing, Xinxiang Medical University, 453000 Xinxiang, Henan, China; 2 Nursing Department of Henan Provincial People’s Hospital, 450003 Zhengzhou, Henan, China

**Keywords:** Late-life depression, Anemia, Suicide attempt, Serum ferritin, Folate, Vitamin B_12_

## Abstract

**Background:**

Anemia is strongly associated with late-life depression (LLD), however, few studies have investigated the relationship between anemia and suicide attempts in LLD patients. It is still challenging to predict suicide risk in patients with depression. Therefore, there is growing interest in potential biomarkers of depressive disorders and suicidal behavior, which may play a significant role in the early diagnosis and treatment of depression. This study aimed to compare serum ferritin, folate, vitamin B_12_, and erythrocyte parameter levels in patients with LLD with those in healthy older adults, and the relationship between serum ferritin, folate, vitamin B_12_, and suicide attempts in patients.

**Methods:**

Serum ferritin, folate, vitamin B_12_, and erythrocyte parameter levels were measured in 66 hospitalized LLD patients (30 without suicide attempt, 36 with suicide attempt) and 47 healthy individuals. All participants were surveyed for basic conditions and suicide attempts, and depression was assessed in LLD patients.

**Results:**

Serum ferritin, folate, vitamin B_12_, red blood cell count, hemoglobin, hematocrit, mean platelet volume and plateletcrit levels were significantly lower in LLD patients compared with healthy older adults (*P* < 0.05). Further analysis of the relationship between serum ferritin, folate, and vitamin B12 levels and LLD patients’ suicide attempts and showed a significant negative association between serum folate and vitamin B12 and suicide attempts (*P* < 0.05).

**Conclusions:**

Serum ferritin, folate, vitamin B_12_, red blood cell count, hemoglobin, hematocrit, mean platelet volume and plateletcrit levels were significantly lower in LLD patients than in healthy older adults. In addition, reduced serum folate and vitamin B_12_ levels in patients may have some effect on suicide attempts. More mechanistic studies are needed to further explain this association.

## Introduction

Late-life depression (LLD) is one of the most serious threats to the mental health of older adults that not only reduces quality of life, but also influences the prognosis of other chronic diseases that further aggravate disability. Compared with younger patients with depression, LLD patients’ symptoms manifest more as a lack of interest, sleep disturbances, mental retardation, and so forth, and are accompanied by extensive cognitive impairment. The incidence of LLD can be as high as 9–45% [1]. A study in Portugal found that the prevalence of depression in older adults was approximately 15.3% [2]. The prevalence of depressive symptoms ranging from mild to major was as high as 35% among community-dwelling older adults in the United States [3]. Zhong et al. [4] revealed that 5.5–5.9% of the community-residing older adults had depressive disorders in China,and as high as 96.1–97.7% of the depressed older adults had never sought any help from mental health specialists.

Additionally, suicide attempts are closely related to LLD. The Centers for Disease Control and Prevention define a suicide attempt as “a non-fatal, self-directed, potentially injurious behavior with an intent to die as a result of the behavior” [5]. It can be interpreted as suicidality falls on a continuum from suicidal thoughts that are associated with no action to suicidal ideation with intent to harm one’s self, and finally, to an actual suicide attempt. Suicide attempts are the strongest precursors of subsequent completed suicides. Approximately 60% of all suicides occur in the context of depressive disorders [6]. Additionally, approximately 10% of people with LLD have attempted suicide. Patients with LLD who have attempted suicide experience more severe depressive symptoms and higher recurrence rates than those who have not [7]. Thus, LLD has become a serious public health problem worldwide, and is the main cause of disease burden and global disability.

Among the risk factors for depression, anemia has received increasing attention. Anemia is a common disorder in older adults, with an estimated global prevalence of 24% in the older adult population [8]. Several studies have shown a significant association between anemia and depression [9]. Anemia is commonly associated with diseases such as cancer, chronic renal failure, and malnutrition, which subsequently lead to reduced quality of life, thereby increasing the risk of depression. Additionally, anemia decreases muscle strength, leading to falls, decreased physical fitness, prolonged hospitalization, and increased mortality [10]. Conversely, depression may contribute to the development of anemia through unhealthy lifestyles, such as alcohol intake or inadequate nutritional intake. Moreover, anemia is more prevalent in patients with psychiatric disorders, including depression, than in the general population [11]. Anemia not only alters red blood cell parameters in patients, but is also associated with changes in serum ferritin, folate, and vitamin B_12_ levels.

Recent evidence suggests that depression is accompanied by biochemical and immune changes and that there is a chronic inflammatory response. Nutrition is strongly associated with the occurrence and development of depression. Previous studies have shown that high plasma concentrations of homocysteine (Hcy) are a potential risk factor for depression, and that folate and vitamin B_12_ can decrease Hcy concentrations [12]. Evidence suggests that lower folate levels are associated with a decrease in cognitive performance, psychomotor speed, and greater depressive symptoms. This is because folate plays an important role in the involvement of methylation processes and proper functioning of the carbon metabolism cycle, which is essential for neurodevelopment and neurological health [13]. Vitamin B_12_ also plays an important role in DNA synthesis and neurological function. Several previous cohort studies, randomized controlled studies, and meta-analyses have shown that low levels of serum folate and vitamin B_12_, as well as low dietary intake of folate and vitamin B_12_, are associated with an increased risk of depression [14]. Iron status also plays an important role in brain function, cognition, and behavior; ferritin, a ubiquitous intracellular protein that stores and releases iron, has been widely used as a clinical marker of iron status [15]. Iron deficiency, usually characterized by decreased in the population, may lead to mental, emotional, and behavioral changes that can lead to mood disorders [16]. However, the results of current research on the relationship between ferritin and depression are controversial. Previous studies have demonstrated a positive correlation between depressive symptoms and low levels of serum ferritin [17]. Conversely, elevated serum ferritin levels have been associated with post-stroke depression in patients with stroke [18]. 

Although most suicides occur in the context of depressive disorders, it is still challenging for clinicians to predict suicide risk in patients with depression. Therefore, there is growing interest in potential biomarkers of depressive disorders and suicidal behavior, which may play a significant role in the early diagnosis and treatment of depression. However, there has been controversy about the association between anemia and suicide attempts. A large-scale pharmacoepidemiologic study of folate found a beneficial association in terms of lower rates of suicide attempts, every additional month of folate treatment was associated with a 5% reduction in the suicidal event rate [19]. And aother indicated that there was no evidence for an association between lower complete blood count parameters and suicide attempt [20]. Although the correlation between anemia and depression in one side and depression and suicide in another side is previously shown in many articles. However, no study has clarified whether an association exists between the levels of ferritin, folate, vitamin B_12_, and suicide attempts. Therefore, this study discusses and analyzes the relevant blood indicators and influencing factors in hospitalized LLD patients.

## Methods

### Study population

**Fig. 1:**
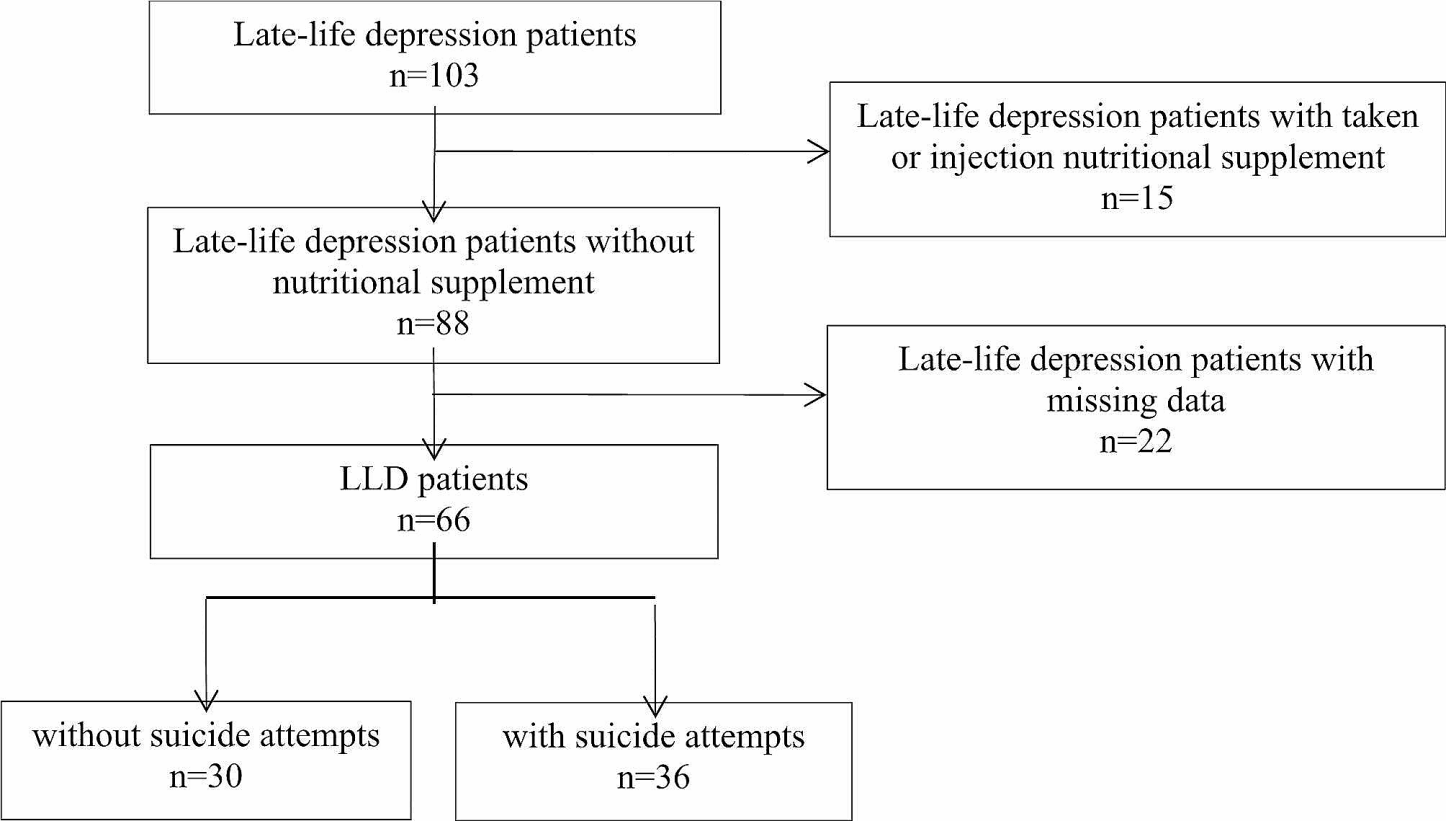
Flow chart of LLD patients. A total of 103 late-life depression patients were investigated, of which 15 subjects with taken or injection nutritional supplement were excluded, and 22 subjects were missing data, finally, 66 late-life depression patients were enrolled. The diagnosis of 30 LLD without suicide attempts and 36 with suicide attempts were made by a professional psychiatrist following a diagnostic criterion of depression according to the International Classification of Diseases version 10 (ICD-10).

This was a case-control study that included 66 LLD patients (30 without suicide attempt, 36 with suicide attempt) and 47 healthy individuals; it was conducted from June to October, 2020, at a psychiatric hospital in Henan Province. The study was approved by the Hospital Ethics Committee (approval no: XYLL—2020251) and informed consent was granted by the patients or their relatives. The inclusion criteria for patients were as follows: (i) age ≥ 60 years and (ii) LLD diagnosis. Exclusion criteria were as follows: (i) a history of severe gastrointestinal disease and gastrointestinal surgery affecting nutrient absorption; (ii) cognitive dysfunction and an inability to clearly express themselves; and (iii) administered nutritional supplements in the past three months (Fig. 1). For our patients recruitment, we considered only patients that required hospitalization. Simultaneously, 47 healthy controls were recruited from physical examinations during the same period, and any individuals with a personal or family history of psychiatric illness were excluded.

### Clinical variables

Basic information about the participants, including their sex, age, education, Body Mass Index (BMI), place of residence, frequency of physical exercise, smoking status, alcohol consumption, co-morbid chronic diseases, and disease duration, were collected via questionnaires. The diagnosis of depression and suicide attempts were made by a professional psychiatrist following a diagnostic criterion of depression according to the International Classification of Diseases version 10 (ICD-10). The Self-Rating Depression Scale (SDS) was used to assess participants’ depressive state.

### Laboratory tests

Fasting venous blood samples (8 ml) were collected from both groups in the morning, then centrifuged at 3000 r/min for 5 min. Vitamin B_12_, folate, and ferritin levels were detected using the electrochemiluminescence immunoassay (ECLIA) method (Cobas 6000, Roche Diagnostics). Blood cell count (red blood cell count) [RBC], hemoglobin [HGB], hematocrit [HCT], mean corpuscular volume [MCV], mean corpuscular hemoglobin [MCH], mean corpuscular hemoglobin concentration [MCHC], platelet count [PLT], mean platelet volume [MPV], and plateletcrit [PCT]) were measured using the blood analyzer Sysmex XN-2000 (Sysmex Corporation, Japan). The experimental procedures were conducted in strict accordance with the kit instructions.

### Statistical analyses

SPSS software version 22.0 was used for data analysis. Categorical variables were analyzed using the Chi-square test and expressed as proportions. Normally distributed variables differences between the two case groups (LLD and LLD with suicide attempt) and control group (healthy subjects) were estimated using ANOVA for numerical variables with an additional Bonferroni post-hoc test and expressed as mean (± SD). Non-normally distributed variables were analyzed using the Mann-Whitney *U* test and expressed as medians (interquartile ranges). Differences were considered statistically significant at *P* < 0.05.

## Results

### Demographical features and SDS scores in the three groups

A total of 113 participants met the inclusion criteria and participated in the study, including 47 in the healthy subjects, 30 LLD without suicide attempt and 36 LLD with suicide attempt. The mean (± SD) age of three groups was 67.30 ± 0.78, 66.37 ± 1.17 and 66.53 ± 0.89 years, respectively. The majority of the participants in three groups were female (61.7%, 63.3% and 61.1%, respectively). The mean (± SD) BMI was 23.62 ± 0.40, 23.62 ± 2.76 and 23.45 ± 3.07, respectively. No significant difference in Demographical features were observed between groups (*P*>0.05). The SDS scores was 32.36 ± 1.71, 61.27 ± 2.13 and 71.75 ± 1.88, respectively, with a significant difference (*P*<0.001) (Table 1).

**Table 1:** Demographical features and SDS scores of three groups

	Healthy subjects, *n* = 47	LLD without suicide attempt, *n* = 30	LLD with suicide attempt, *n* = 36	F/x^2^value	*P* value
Age (years)	67.30 ± 0.78	66.37 ± 1.17	66.53 ± 0.89	0.313	0.732
Sex (Female, %)	61.7	63.3	61.1	0.018	0.982
BMI (kg/m^2^)	23.62 ± 0.40	23.62 ± 2.76	23.45 ± 3.07	1.708	0.186
SDS Scores	32.36 ± 1.71	61.27 ± 2.13	71.75 ± 1.88	129.522	<0.001^*†‡^

^*^Statistically significant difference between Healthy subjects and LLD with suicide attempt

^†^Statistically significant difference between LLD without suicide attempt and LLD with suicide attempt

^‡^Statistically significant difference between Healthy subjects and LLD without suicide attempt

### Serum ferritin, folate, and vitamin B_12_ levels in two case groups were significantly lower than that in the control group

Post-hoc analysis between the two groups comprised of patients with LLD with suicide attempt (*P* < 0.05) and without suicide attempt (*P* < 0.05) showed significantly lower Serum ferritin, folate, and vitamin B12 levels compared to the control group. Folate were lower in the LLD without suicide attempt group with respect to the control group (*P* < 0.05). Also, patients with LLD who were associated with suicide attempt had significantly lower folate and vitamin B12 compared with LLD without suicide attempt (*P* < 0.05) (Table 2).

**Table 2:** Ferritin, folate and vitamin B_12_ of three groups

	Healthy subjects, *n* = 47	LLD without suicide attempt, *n* = 30	LLD with suicide attempt, *n* = 36	*Z* value	*P* value
Ferritin (mL/ng)	154.10(127.80-227.10)	142.75(78.60-182.08)	126.45(71.71-158.25)	8.529	0.014^*^
Folate (ng/mL)	9.20(7.11–12.63)	7.02(3.67–9.89)	4.82(3.69–6.11)	33.288	<0.001^*†‡^
Vitamin B_12_ (pg/mL)	445.00(361.00-610.00)	367.55(238.93-617.23)	280.35(214.90-378.23)	24.552	<0.001^*†^

^*^Statistically significant difference between Healthy subjects and LLD with suicide attempt

^†^Statistically significant difference between LLD without suicide attempt and LLD with suicide attempt

^‡^Statistically significant difference between Healthy subjects and LLD without suicide attempt

### RBC, HGB, HCT, MPV and PCT levels in LLD patients were significantly lower than the control group

RBC, HGB, HCT, MPV and PCT were lower in the LLD without suicide attempt group with respect to the control group (*P* < 0.05). HGB, HCT and MPV were lower in the LLD with suicide attempt group with respect to the control group (*P* < 0.05). But two case groups no significant association was found (*P*>0.05) (Table 3).

**Table 3:** Complete blood count parameters and other biochemical analyses of three groups

	Healthy subjects, *n* = 47	LLD without suicide attempt, *n* = 30	LLD with suicide attempt, *n* = 36	*Z/F* value	*P* value
RBC (10^12/L)	4.56 ± 0.06	4.27 ± 0.79	4.34 ± 0.96	3.677	0.028^‡^
HGB (g/L)	140.60 ± 1.80	132.53 ± 2.41	132.03 ± 2.67	5.001	0.008^*‡^
HCT	0.43 ± 0.01	0.40 ± 0.01	0.40 ± 0.01	4.327	0.016^*‡^
MCV (fL)	93.40(91.40–95.20)	94.30(92.55–96.53)	94.20(91.23–96.40)	1.661	0.436
MCH (pg)	30.80(30.10–32.10)	30.70(30.20-31.75)	30.90(29.65–31.75)	1.431	0.489
MCHC (g/L)	330.64 ± 1.08	328.53 ± 1.43	326.58 ± 2.11	1.874	0.158
PLT (10^9/L)	236.00(214.00-259.00)	234.00(188.25–271.00)	218.50(193.75–257.50)	0.658	0.719
MPV (fL)	9.85(9.30–10.50)	7.10(6.58–8.83)	7.55(6.60–9.50)	32.345	<0.001^*‡^
PCT	0.23(0.18–0.25)	0.17(0.14–0.19)	0.18(0.13–0.23)	13.477	0.001^‡^

^*^Statistically significant difference between Healthy subjects and LLD with suicide attempt

^†^Statistically significant difference between LLD without suicide attempt and LLD with suicide attempt

^‡^Statistically significant difference between Healthy subjects and LLD without suicide attempt

## Discussion

This study aimed to compare serum ferritin, folate, vitamin B_12_, and erythrocyte parameter levels in LLD patients with those in healthy older adults, and the relationship between serum ferritin, folate, vitamin B_12_, and suicide attempts in the patients. A large national survey of older adults found a significant association between anemia and depressive symptoms after adjusting for age, sex, occupation, vitamin supplement intake, smoking status, and BMI [21], which was confirmed by another cross-sectional studies [22]. 

Serum ferritin is an indicator of iron levels in the body. Iron is involved in physiological activities, such as mitochondrial oxidation reactions and hemoglobin synthesis, as an electron transmitter. It is also involved in nerve myelin production and the synthesis of myelin and neurotransmitters, which is important for functional brain activity, and deficiency can lead to iron deficiency anemia. In addition, vitamin B_12_ and folate, which are essential nutrients in organisms, are also involved in carbon metabolism during the synthesis of several monoamine neurotransmitters and are associated with megaloblastic anemia and neurological disorders [23]. 

In our study, serum ferritin levels were significantly lower in LLD patients compared to healthy controls. This result is identical to the findings of another study, [24] the study measured serum HGB, ferritin, transferrin receptor levels, and depressive symptoms in 1,802 older adults aged 65 years and older who participated in the Korea Health Survey. Serum ferritin deficiency usually coincided with depressive symptoms in older adults, which was confirmed by another cross-sectional study [25]. However, some studies have presented different results. For example, elevated serum ferritin levels was found in a serum proteomic study in patients with major depression [26]. In contrast, a cross-sectional study of 3,839 adults in China, no significant association between serum ferritin levels and depressive symptoms [27]. These contradictory results may be not to evaluate participants for inflammatory diseases, iron or vitamin supplement use, and other medications that may affect serum ferritin levels, all of which may contribute to increased ferritin levels.

In addition, folate and vitamin deficiencies are common among older adults and may be attributed to malnutrition or malabsorption. SDS is one of the Ministry of Health and Welfare Psychopharmacology recommended by the American educational institute, which can assess the severity of depressive symptoms, the higher the SDS score, the more severe the depression was. Our study findings showed a significant negative correlation between folate levels and depressive symptoms. This result is similar to that of another study, [28] which found that depressed patients had significantly lower folate levels than those without depression and had lower folate intake compared to non-depressed patients. Furthermore, depressed patients with low folate levels were less sensitive to antidepressant treatment and were more likely to relapse. In contrast, adequate intake of folate was demonstrated as a protective factor against the continued development of depressive symptoms. Moreover, patients with low serum folate levels improved their depressive symptoms for a longer mean time (3.5 versus 5 weeks) than did those with normal folate levels [29]. Therefore, serum folate levels should be assessed in all patients receiving treatment for depression, and timely use of folate supplements may improve clinical outcomes in depressed patients. The positive association between low serum folate levels and depressive symptoms detected in the present study is consistent with earlier studies; however, earlier studies found no association between vitamin B_12_ and depressive symptoms [14]. Current findings showed that serum vitamin B_12_ levels were significantly lower in LLD patients compared with healthy older adults. Another study showed that low serum vitamin B_12_ concentrations and decreased vitamin B_12_ concentrations in older adults were predicted depression [30]. In vegetarians in the United Kingdom, Berkins et al. found that particularly those who suffer from depression, may benefit from supplementing their diets with vitamins B6, B12, and folate to prevent loss of brain volume and to ensure better mental health [31]. The relationship between vitamin B_12_ and depression is unclear, and it may be related to differences in vitamin B_12_ intake or study methods in the investigated populations.

RBC, HGB, HCT, MPV, and PCT levels were lower in older patients with LLD than in healthy controls. This is consistent with previous studie that demonstrated an association between major depression and decreased erythrocyte parameters. Low erythrocyte parameters may be a risk factor for depression. A possible explanation for the lower erythrocyte parameters in patients with depression is that in major depression, the immune-inflammatory response may induce iron metabolism and erythrocyte disturbances.

We assessed the relationship between suicide attempts and ferritin, folate, and vitamin B_12_, which had not been comprehensive assessed in other studies. Our results showed that serum folate and vitamin B_12_ levels were significantly and negatively associated with suicide attempts in older patients with depression, whereas serum ferritin levels were not significantly associated with suicide attempts. A recent study found reduced serum folate levels (< 6.0 ng/mL) were independently associated with all four types of suicidal behaviors [32]. Another study demonstrated that those patients with a family history of suicide showed significantly lower levels of vitamin B12, as compared with the rest of the sample [33]. We believe that these results justify advocating for an RCT to study the effect of serum folate and vitamin B_12_ on suicidality. More mechanistic studies are needed to further explain this association.

## Limitations

Nevertheless, the present study has several limitations. First, our study did not test for homocysteine (Hcy), which is a thiol group containing the amino acid which naturally occurs in all humans. Hyperhomocysteinemia (HHcy) can be caused by deficiencies in the levels of folate and vitamin B_12_. Therefore, this study should be followed up by a detection and analysis of Hcy levels in LLD patients. Additionally, we did not assess dietary intake and cannot exclude the possible effects of dietary habits on blood indicators. However, the study participants were from the same region and had the same cultural and economic backgrounds; therefore, it was assumed that their dietary habits are similar. Finally, we used the SDS to assess the severity of the patients’ disease, and the results may be subjectively influenced by the patients. In the future, it is important to deeply analyze the contradictory results of anemia-related indicators in LLD. This will help to verify whether hematological indicators can be used as biochemical factors for the early identification of older adults at risk of depression. These findings can be further be used as important indicators for the prevention of LLD and to provide scientific guidance for enhancing the mental health of older adults.

## Conclusion

In conclusion, our study showed that serum ferritin, folate, vitamin B_12_, red blood cell count, hemoglobin, and hematocrit levels were significantly lower in LLD patients than in healthy older adults, and that folate and vitamin B_12_ levels may have some effect on suicide attempts in patients. If this finding is validated in additional studies, it could be used as a preventive strategy for depression in the elderly. Measuring hematological indicators serves as a minimally invasive and simple method that clinicians can use in conjunction with clinical symptoms in older adults for timely diagnosis and early intervention.

## Data Availability

The datasets analyzed in the current study are available from the corresponding author on reasonable request.

## Abbreviations

LLD: Late-life depression
Hcy: Homocysteine
BMI: Body Mass Index
ICD-10: International Classification of Diseases version 10
SDS: Self-Rating Depression Scale
ECLIA: Electrochemiluminescence immunoassay
RBC: Blood cell count (red blood cell count)
HGB: Hemoglobin
HCT: Hematocrit
MCV: Mean corpuscular volume
MCH: Mean corpuscular hemoglobin
PLT: Platelet count
MPV: Mean platelet volume
MCHC: Mean corpuscular hemoglobin concentra-tion
HHcy: Hyperhomocysteinemia
PCT: Plateletcrit
